# Understanding the quality‐of‐life experiences of older or frail adults following a new dens fracture: Nonsurgical management in a hard collar versus early removal of collar

**DOI:** 10.1111/hex.14017

**Published:** 2024-03-15

**Authors:** Mia Closs, Paul Brennan, Angela Niven, Susan Shenkin, Helen Eborall, Julia Lawton

**Affiliations:** ^1^ Usher Institute The University of Edinburgh Edinburgh Scotland; ^2^ Centre for Clinical Brain Sciences The University of Edinburgh Edinburgh Scotland

**Keywords:** cervical collar, dens fracture, older adults, patient perspective, qualitative research, quality‐of‐life

## Abstract

**Introduction:**

In the United Kingdom, fractures of the cervical dens process in older and/or frail patients are usually managed nonsurgically in a hard collar. However, hard collars can lead to complications and this management approach is now being questioned, with growing interest in maximising patients' short‐term quality‐of‐life. It is vital that patients' perspectives are considered; yet, there is a dearth of literature examining the aspect. To help inform wider decision‐making about use of collar/no collar management of dens fractures in older/frail people, we explored older/frail people's experience of the two management approaches and how they affected their perceived quality‐of‐life.

**Methods:**

We interviewed older and/or frail adults with a recent dens fracture (aged ≥65 years or with a clinical frailty score of ≥5) or their caregiver. Participants were recruited from both arms of a clinical trial comparing management using a hard collar for 12 weeks (SM) with early removal of the collar (ERC) and were interviewed following randomisation and again, 12–16 weeks later. Data were analysed using a framework approach.

**Results:**

Both participant groups (SM/ERC) reported substantial, negative quality‐of‐life (QoL) experiences, with the fall itself and lack of access to care services and information being frequent major contributory factors. Many negative experiences cut across both participant groups, including pain, fatigue, diminished autonomy and reduced involvement in personally meaningful activities. However, we identified some subtle, yet discernible, ways in which using SM/ERC reinforced or alleviated (negative) QoL impacts, with the perceived benefits/burdens to using SM/ERC varying between different individuals.

**Conclusion:**

Study findings can be used to support informed decision‐making about SM/ERC management of dens fractures in older/frail patients.

**Patient or Public Contribution:**

Public and patient involvement contributors were involved in the study design, development of interview topic guides and interpretation of study findings.

## 1 INTRODUCTION

Fractures of cervical vertebrae, including fractures of the second cervical vertebra (dens fractures), are increasing in frequency, occur predominantly in older (over 65 years) or frail people and mostly result from low‐velocity falls.^1^, ^2^, ^3^, ^4^, ^5^ Due to increased surgical risk and poor bone quality, older and/or frail patients in the United Kingdom are usually managed nonsurgically with external immobilisation in a hard collar.^6^, ^7^, ^8^ External immobilisation aims to provide cervical stability to promote bony fusion, prevent neurological deterioration and minimise short‐ and long‐term pain.^7^, ^9^ However, hard collars can lead to complications, including pressure sores and pneumonia.^10^, ^11^, ^12^ They may also cause difficulties with swallowing and affect the ability to undertake routine activities of daily living, including self‐care.^13^, ^14^

Due to these potential problems, and because bony union is not considered necessary for acceptable clinical outcomes in older and/or frail people, management of dens fractures using hard collars is now being questioned in this population.^15^ Additionally, high mortality rates in older and/or frail people in the first year following a dens fracture^5^ have prompted suggestions that it may be more important to maximise short‐term quality‐of‐life (QoL) than long‐term radiological outcomes. Hence, there is a growing interest in managing these patients without any immobilisation and growing emphasis upon basing treatment decisions on QoL rather than clinical considerations per se.^15^, ^16^

While studies have examined clinical and radiological outcomes of the different management options for dens fractures, there is a dearth of research exploring patients' perspectives and experiences during treatment with or without a hard collar. Understanding patients experiences has long been accepted as playing an important role in improving healthcare quality.^17^, ^18^ There are often discrepancies between clinicians and patients perceptions of, and priorities for, health care at both an individual and group level and, given the importance attached to maximising QoL in older and/or frail people with dens fractures, it is vital that their experiences and perspectives are considered.^17^, ^18^ To address this important gap in the literature, we undertook an interview study to understand and explore how using/not using hard collar management affected older and/or frail people's everyday QoL. Our objectives were to help inform decision‐making about using hard collar/no collar management in older and/or frail people who have dens fractures where, increasingly, and due to the limited life expectancy in this particular patient population, optimising QoL is considered the paramount priority.

## 2 METHODS

This qualitative study was embedded within a clinical trial that sought to test the hypothesis that early removal of the hard collar (<3 weeks following the injury) would improve QoL outcomes (measured using the EQ‐5D‐5L) in older and/or frail patients, compared to standard nonsurgical management with a hard collar.^16^ The trial is registered with the US National Library of Medicine (ref: NCT04895644).

To be eligible for the trial, individuals needed to have been diagnosed with a recent (<3 weeks) dens fracture and be either ≥65 years old or have a Clinical Frailty Scale (CFS) of ≥5 (the CFS is widely used in the United Kingdom to inform treatment decisions for frail and/or older patients who present in Emergency Departments with dens fractures).^19^ Frailty is a contested construct with multiple interpretations,^20^ and various definitions and clinical assessment tools exist. In line with the use of the CFS within the wider trial, our use of the term relates specifically to the CFS and the concept of deficit accumulation.^21^, ^22^ However, by using the term in this way, It is important to acknowledge that study participants clinically defined as ‘older’ or ‘frail’ may not necessarily have thought of themselves as being so.^23^, ^24^

Ethical review for this study was obtained as part of the trial application (REC ref: 21/SS/0036—Scotland; REC ref: 21/YH/0141—England). Reporting of this qualitative study conforms to the standards for reporting qualitative research recommendations.^25^

### 2.1 Participants and recruitment

Recruitment took place in seven trial sites across Scotland and England.  Patients (trial participants) with the capacity to consent were recruited to the qualitative study by trial staff at the same time as they were recruited and consented into the trial. When trial staff determined that patients lacked capacity, their primary caregiver was invited to participate in the interview study instead. In some instances where patients agreed to take part in an interview, their primary carer also expressed a wish to be involved. In these instances, a joint interview was undertaken to allow both individuals opportunities to share their perspectives and experiences.

Participants were recruited from both trial arms to understand the ways in which management allocation (standard nonsurgical management in a hard collar [SM]/early removal of collar [ERC]) impacted their everyday, QoL experiences.  Purposive sampling was used to attain a diverse sample with respect to age, location (including local deprivation, rurality, ethnic diversity, living at home/care home), CFS, capacity/incapacity and comorbidities.

### 2.2 Data collection

Participants were interviewed by telephone at two‐time points: shortly after randomisation and 12–16 weeks later.  A longitudinal approach was chosen to allow us to rapidly capture participants' perceptions of their QoL before the fracture, in order to then be able to better understand and explore their QoL experiences over the course of their SM or ERC.^26^, ^27^

The baseline and follow‐up interview guides were developed in light of literature reviews (including the experience of trauma injury in older people) and inputs from clinical coinvestigators and public and patient involvement representatives. They were also revised in light of emerging findings. Throughout, the interviewer used broad, open‐ended questions and follow‐up lines of enquiry to allow individuals' experiences to be probed and explored in depth.  Baseline interviews explored participants' general health, lifestyle/activities and QoL before their fracture, as well as their experience of the fall and understanding of their diagnosis and allocated management. Follow‐up interviews examined participants' experiences since the fracture/first interview, the perceived impact of SM/ERC on everyday activities and QoL and issues around adherence to allocated management. The interview guides are provided in the appendices.

While specific definitions of QoL vary greatly between different disciplines, reviews across the literature suggest that QoL is a highly individual, multidimensional and context‐specific experience^28^, ^29^ and this interpretation informed our exploration of QoL issues during data collection.

Interviews took place between December 2021 and April 2023 and lasted 25–80 min. All were digitally recorded, transcribed in full and checked for accuracy.

### 2.3 Analysis

Data analysis was undertaken by three experienced qualitative researchers (MC, HE, JL). This analysis was guided by the Framework approach, in particular, the works of Ritchie and Spence,^31^ Smith and Firth^32^ and Gale et al.,^30^ due to the volume and complexity of the data collected. We began with an initial period of data immersion, wherein all three members of the research team repeatedly read through and cross‐compared interviews before bringing their preliminary interpretations together. Preliminary interpretations were found to be very similar and broadly aligned with the literature on QoL in older adults, specifically, the findings reported in Van Leeuwen et al.'s^29^ recent thematic synthesis of the qualitative literature on QoL experiences in older adult populations. Hence, a decision was made to use the domains identified in this synthesis, as part of a theory‐informed method, to guide and refine the next stage of the analysis, as this allowed a comprehensive analytical approach to be maintained at the same time as providing an evidence‐based framework to guide the work.

The framework comprised nine QoL domains: *autonomy; role and activity; health perception; relationships; attitude and adaption; emotional comfort; spirituality; home and neighbourhood*; and *financial security*.^29^ All interviews were coded using these nine domains and coded data sets were subjected to further, in‐depth analyses to allow more granular interpretations of the data to be developed and illustrative quotations identified. A key element of the analysis at this stage involved cross‐comparing the perspectives and experiences of participants in the SM and ERC groups to identify QoL issues that cut across the two participant groups (and the reasons for these) as well as those that were more specific to participants within each treatment group.

Charting the data in full was undertaken by the first author (MC), with regular peer scrutiny throughout the analysis process. To avoid the issues of ‘analysis by committee’,^33^ (MC) initially conducted mapping and interpretation, with (JL) and (HE) challenging and checking the interpretations made. Differences in interpretation and coding were resolved easily through discussion.

The qualitative software package NVIVO 20 was used to store the data and facilitate data coding and retrieval.

## 3 RESULTS

### 3.1 Participants

Thirty‐two individuals were interviewed in relation to the experiences of 27 trial participants. Fourteen had been randomised to SM and 13 to ERC. Trial participant ages ranged from 64 to 94 years, and their CFS ranged from 1 to 7. Participant characteristics are presented in Table 1.

**Table 1:** Participant group characteristics.

Age	64–94 years. Median: 82 years, mode: 78 years.
CFS	Median score: 4, mode: 6.
Trial allocation	13 Patients allocated to ERC, 14 patients allocated to SM.
Place of residence preaccident	26 Patients lived at home, six of whom lived alone, 19 with their spouse/partner and one with their adult off‐spring.
	1 Lived in a nursing home.
Capacity	2 Patients were adults with incapacity.
Support needs prefracture	17 Patients were independent in ADLs.
	5 Required support with a few ADLs.
	5 Required support with many or most ADLs.
Interviewee	16 Interviews with patient alone.
	6 With proxy alone, including three spouses/partners and three adult off‐spring.
	5 With both the patient and their proxy.
Reasons for proxy interview	Dysphasia, hearing difficulties, ongoing short‐term memory loss, ongoing pain or fatigue related to dens fracture, advanced dementia (adults with incapacity).

Abbreviations: ADL, activities of daily living; CFS, Clinical Frailty Scale.

Five participants did not take part in follow‐up interviews: one withdrew from the trial, one died, one could not be contacted and two declined further interviews for health‐related reasons.

### 3.2 Quality‐of‐life experiences

Both groups of participants (SM/ERC) reported significant QoL impacts over the course of the fracture management. These related to almost all QoL domains reported by Van Leeuwen et al.,^29^ with the fall itself being a contributory factor in some instances. Below, we discuss QoL impacts that cut across the groups as well as highlighting those more specific to each treatment group. Participants seldom talked about experiences relating to the QoL domains of *spirituality*, or *home and neighbourhood*; hence, these are not included in the reporting below. *Financial security* was only raised in relation to aspects of *attitude and adaption* and so these domains are discussed together. Additional quotes and illustrative data are available in the appendices.

#### 3.2.1 Autonomy

Many participants reported already living with significant comorbidities and frailties and, as a consequence, requiring help with basic activities of daily living before their fall/fracture. Additionally, some, especially those with high CFS scores, were, at the time of the accident, either living in a care home or receiving full‐time care at home. For such individuals, the dens fracture was often presented as a minor issue compared to all the other health conditions already affecting their autonomy and QoL:

[NAME] … has dementia. He has heart failure, he has osteoporosis, leukaemia, prostatitis, glaucoma, you name it … he's got lots of health issues. So the neck has … not been an issue at all, it's all his other problems. (D1_SM)

Nonetheless, all participants did report experiencing decreased autonomy following the fracture, regardless of the presence/absence of pre‐existing frailties or comorbidities or their allocated treatment. For example, all described needing some/increased help with bathing and dressing, shopping, cooking and laundry. In doing so, some discussed mindfully restricting what help they asked for or allowed family members to give to avoid becoming a burden to others.

SM participants reported additional impacts on their autonomy resulting from use of the collar. For example, they often reported needing help with feeding (e.g., having food cut up or fed to them) after missing their mouths and spilling food and drinks down their collar.

Obviously you can't bend your neck forwards very much. And because she couldn't see very well, she found eating difficult. So we used to go in and actually feed her. (D33_SM)

These individuals also described difficulties going downstairs without help as the upright positioning of their head made it difficult to look down and place their feet correctly on the steps. Furthermore, some SM participants interpreted advice from the hospital to keep the collar dry and not remove it as strict instructions. In the absence of help from any health or social care professionals, some described giving up washing or styling their hair, shaving or showering for the entire fracture management period and limiting themselves to freshening up with a flannel instead.

#### 3.2.2 Role and activity

Participants described a variety of day‐to‐day activities that they/the patient had enjoyed before the accident that had kept them busy, in touch with others and given them a sense of purpose, although these tended to be most frequently vocalised by those with lower CFS scores, who also tended to report fairly high degrees of physical autonomy and independence before the fall. Such participants described how these activities had been compromised by the fracture, due to localised and general fatigue, pain, loss of confidence and anxiety.

I've just not had the strength or the energy to be honest. And I didn't want to fall. Like my main thing, I mean I've not been down the back garden since [the fall in the garden] …. It's funny, it makes you lose your confidence … And I love going to feed the birds. (D16_ERC)

Specifically, participants highlighted problems undertaking activities such as reading, craftwork, woodwork and snooker due to experiencing difficulties with neck movements. While some attributed these difficulties to the collar restricting head movement, individuals randomised to ERC described how neck pain could also limit their ability to look up or down. Additionally, those allocated to SM reported difficulties wearing hearing aids while using the collar, which led to feelings of disconnection, difficulties communicating and reduced socialisation. Several participants also reported feeling self‐conscious wearing the collar in public, which led some to withdrawal from social activities.

#### 3.2.3 Relationships

The importance and appreciation of close and caring relationships were evident throughout all accounts, with some participants noting how the normal division of labour within their marriage or associated with being a carer for a dependant family member was altered or reversed by the fracture. In some instances, this meant that the care recipient had now become the caregiver.

I mean me husband he's really wobbly on his legs with this balance problem, so I have to be his carer. But he's caring for me at the moment. So I don't know what'll happen, but we're a'right. (D16_ERC)

Participants in both groups discussed how family and friends became protective following the fracture. While the collar was reported as being a constant reminder to others of the individual's vulnerability and discomfort, ERC participants also noticed that family/friends acted in protective ways due to their concerns about the injury healing without protection of a collar.

The availability of healthcare support varied widely, with many participants highlighting widespread staffing difficulties within the National Health Service (NHS) and social care sectors at the time of their interviews. When care services were available, participants often described staff positively, stating ‘they're very good. I can't fault them’ (D39_ERC). However, across both treatment groups, many participants reported a paucity of help, support, guidance and advice, even when specifically requested.

I mean, it was quite difficult. The Monday after he did it I went to our GPs and asked them … ‘cause he needed a frame’ cause the stick wasn't strong enough … And I must admit, I broke down there because all they said to me was, go to social services. I'phoned social services and they said to me, you're on a list. And that was it. (D6_ERC)

Although disappointment was common to both groups, SM participants often experienced this in heightened ways. Having been advised in the hospital that the collar pads would be changed every week by their district nurse or physiotherapist, and that they must not remove the collar themselves, participants reported feeling isolated and let down when no one came to help, and no appointments could be made to have this done.

We asked the local surgery to come and put a new liner in … but they didn't come … absolutely nobody [came]. We'd gone to the surgery, we contacted all sorts of people, we even went through [charity working with older people]. Finally [charity] got her a nurse. … And the nurse walked in and said: where's your wound. … She saw the collar and said: oh I'm not touching that, and left. (D10_SM)

In comparison, the minority of SM participants who had regular appointments or had been given training and support reported feeling confident, cared for and reassured that further help was available if they encountered problems.

#### 3.2.4 Emotional comfort

Interviewees' accounts contained multiple references to negative emotions. These included feeling/being ‘worried’, ‘frustrated’, ‘depressed’, ‘distressed’, ‘embarrassed’, ‘frightened’, ‘devastated’, ‘trapped’, ‘afraid’, ‘isolated’, ‘fed up’, ‘bored’, ‘annoyed’, ‘pissed‐off’, ‘tearful’ and ‘upset’. Many of these negative emotions related to the sudden and unexpected nature of the original accident and participants' resultant, heightened awareness of their vulnerability. Participants also described worrying about whether the fracture would heal and being dependent on others for care/assistance, especially in cases where they had had limited frailties before the fall. Those who were caregivers themselves further reported worrying about meeting their dependents' needs in the short and longer term. For participants who were already living with existing frailties or comorbidities, the perceived increase in disability, dependency and pain resulting from the fall/fracture was described as heightening pre‐existing feelings of anguish and distress.

He's really upset with himself, he keeps crying' cause he's really upset with himself for being like he is. And I think he's just fed up with being in pain. (D6_ERC)

Participants further described how the lack of information or advice that they were given on what they could and could not do during their fracture management period could cause significant irritation, frustration and distress. Faced with a lack of clarity, many described limiting both general household activities and hobbies, which further heightened feelings of isolation and upset.

For some SM participants, the collar itself could contribute to their emotional discomfort. For example, some collar users described the indignity of being unable to shave or wash their hair. The tight fit of the collar and complete restriction of head movement also led to some individuals feeling frightened and trapped. Indeed, some participants referred to the collar as a ‘cage’ (D14_ERC). However, for others, the collar appeared to have a positive emotional impact; specifically, participants described feeling ‘safe’ (D1_SM) or ‘cosy’ (D31_SM) by virtue of the collar offering emotional security and physical support. In line with these latter experiences, some ERC participants described worrying about whether removing the collar early would be safe and effective:

My main concern was, you know, this is research, I don't want to damage my health any more than it's already damaged. And envisaging myself with a broken bone in my neck without support was a little scary. (D2_ERC)

#### 3.2.5 Attitude and adaption and financial security

Participants in both treatment groups described a range of practical adaptions to their homes following their accident, including the installation of handrails, raised toilets, bed rails and chair raisers, shower chairs, portable commodes and electric beds. Similarly, some also discussed adapting to how they lived in their homes, such as having their bed moved downstairs or living entirely in one room. Rather than presenting these adaptions in negative ways, many focused on the benefit that these afforded: feeling safer and being able to be at home.

I—I've got two commodes. I've got a commode upstairs for the middle of the night. And I've got a commode downstairs so I don't have to negotiate the steps every time I want to go for a wee. (D34_SM)

While participants only occasionally discussed financial issues, they did so in relation to adaptations when health or social services were not available, acknowledging the benefits to having the financial resources to pay for these kinds of things themselves.

To adapt to living with their fracture and reduce reliance on others, some SM participants described swapping pullovers and T‐shirts for button‐up shirts and V‐neck jumpers or cardigans. These adaptions were, again, presented as positive solutions rather than issues or problems. Similarly, some reported using straws to help drink without spilling and tissues and napkins tucked between their chin and the collar to catch any drips or mess while eating. In addition, while a minority of SM participants described sticking strictly to their treatment allocation, many reported choosing to remove the collar when washing, eating or resting in a supportive chair/sleeping. Participants described their decisions to do so as sticking to the principle of their management allocation, while allowing themselves respite from negative experiences, such as itching, sweating and feeling trapped.

I hate it. Absolutely hate it (laughs) … But like now, I'm not wearing it, because I'm just sitting in the chair, just relaxing. But if I get up and do things, I‐ I put it on. … and I don't sleep in it either eh, I usually get dressed first and then put the collar on. If I'm in the washing machine and, you know, cleaning and things like that, I tend to you know, to put it on. (D38_SM)

Although ERC participants still reported pain and discomfort, many downplayed these experiences by comparing them to the frustration and additional discomfort that they had experienced when they had initially worn their collar. Indeed, using early collar experiences as a comparison, they described their experiences without the collar as ‘not so bad’ (D22_ERC). Hence, the majority of ERC participants did not express a motivation or need to reinstate the hard collar even temporarily, except during the initial 2–3 days when they were first weaning themselves off it.

#### 3.2.6 Health perception

When participants talked about their health following the fracture, they described pain or stiffness in the neck and associated use of medication. Overall, any reduction in pain, analgesic use, stiffness or fatigue was interpreted positively as an indicator of healing.

It's definitely on the mend. It's not as sore as it was. … Before when it was—when I first did it [the fracture], it was really painful. But it's bearable now. (D21_ERC)

There were few apparent differences in the health perceptions of ERC and SM participants, with the role of the collar in pain management appearing to be very individual. While some participants discussed experiencing great pain relief from the collar, others described the pain and discomfort of the collar as being as bad as the pain from the injury itself. Conversely, some participants reported no difference in their pain experiences with or without the collar. Important health‐related QoL considerations emerged with regard to experiences of pressure sores and rashes. Participants who used a hard collar often reported problems affecting their shoulders, upper chest, chin and ears—all areas where the edge of the collar rested. These wounds were described as painful and as adding to participants’ overall discomfort.

## 4 DISCUSSION

Participants described a wide range of experiences that affected their QoL following their dens fracture. Many experiences cut across both participant groups (SM/ERC), including pain, fatigue, diminished autonomy and reduced involvement in personally meaningful activities; experiences were also influenced and informed by the extent to which participants were already experiencing exiting frailties and comorbidities before the fracture. However, SM participants also reported additional issues resulting from wearing the collar, including problems with grooming and maintaining personal hygiene, rashes and pressure sores, challenges retaining a sense of dignity when eating and drinking and difficulties wearing hearing aids, which could amplify feelings of social isolation. Conversely, ERC participants described localised muscle fatigue, concern and uncertainty about undertaking activities during the healing process and heightened feelings of vulnerability without the protection of the collar, leading to reduced activity and social involvement.

In keeping with participants' cross‐cutting experiences, quantitative studies of dens fracture management (surgical and SM) have shown that older and/or frail patients can experience considerable pain, functional loss and an associated decrease in QoL during fracture management.^34^, ^35^ Similar findings have also been reported in studies involving older and/or frail populations following a fall (with or without a fracture) or who experience other types of fracture.^36^, ^37^ Indeed, participants in our study attributed many of their negative, QoL experiences to the physical and psychological impact of their fall and/or subsequent fracture rather than to the use of SM/ERC treatment per se. Nonetheless, our findings highlighted some subtle, yet discernible, ways in which using SM/ERC to manage a dens fracture can reinforce or alleviate (negative) QoL experiences. Our findings further suggest that the QoL impacts of SM versus ERC can vary from one individual to another depending on their personal circumstances (e.g., caregiving responsibilities, availability of family support) and their pre‐existing health conditions and frailties. Thus, this study underscores the importance of presenting individuals with information about all the potential advantages/disadvantages of SM/ERC management so that they can make informed treatment decisions aligned to their personal preferences and circumstances.

The study findings illustrated episodes where (lack of) health and social care availability, home support, resources and pre‐existing frailties or comorbidities interacted with the SM/ERC to adversely affect participants' QoL. When interpreting such findings, it needs to be considered that data collection took place in the early phases of the severe acute respiratory syndrome coronavirus 2 pandemic with rolling national and local lockdowns, a sudden shift from face‐to‐face to telephone consultations and extensive NHS staff burnout and health and social care staff shortages.^38^, ^39^ It is not our intention to criticise staff involved in our participant' care. However, it is important to emphasise the importance of adequate care provisioning being put in place to optimise QoL experiences in this older/frail patient group whether they use SM/ERC treatment or not.

Access to information has been associated with impacts on multiple domains of QoL in older adults.^40^ In keeping with this finding, participants in our study highlighted various ways in which their QoL had been affected by (a lack of) information about activities that were safe or beneficial to undertake during the fracture management period, although the exact nature of the impact on QoL varied between those allocated to SM/ERC. The few participants (SM and ERC) who had been given written information or training before leaving the hospital reported notably different (more positive) experiences. These observations highlight the importance of both SM and ERC patients being given clear and accessible information to support recovery, independence and rehabilitation. To this end, simple, concise, consensus advice about collar care and self‐care, agreed nationally by spinal associations, alongside systems and procedures that ensure that all patients receive such information, may be of benefit while research on optimal dens fracture management is ongoing.

Despite their benefits, assistive devices and home adaptions are often delayed or declined by older adults because of perceived associated stigma.^41^, ^42^ However, participants in this study reported adaptations in a positive light and appreciating the ability to remain at home and be relatively independent. Nonetheless, system barriers to accessing adaptions and equipment (long waiting lists and lack of information/guidance) were experienced by some participants, with resultant, negative impacts. Our findings support ongoing arguments for improved access to services and equipment as well as better collaboration between the organisations working with older adults living at home.^41^, ^42^

Although SM participants reported a greater number of unique impacts on their QoL, this does not necessarily translate into a substantively ‘worse’ overall QoL compared to ERC participants. All of our participants discussed multiple impacts on the same domains of QoL. The findings of this study do not, in themselves, support one management option (SM/ERC) over the other but offer insights that can help guide care provision, provide context for interpretation of the wider literature base and identify areas of unmet need while optimal dens fracture management in the older/frail population is still under investigation.

### 4.1 Strengths and limitations

To the best of our knowledge, this is the first qualitative study to explore frail and/or older people's experiences of using SM/ERC to manage a dens fracture and, hence, to provide an in‐depth understanding of the QoL impacts of both management approaches. However, as study participants were enroled in a randomised clinical trial  and opted‐in to the qualitative research, we may have accessed atypical/highly motivated individuals. Nonetheless, we were successful in interviewing a socioeconomically (although not particularly ethnically) diverse group of individuals. As participants received their treatment as part of a randomised trial, their accounts may have been influenced by therapeutic misconception (i.e., a belief that their randomised treatment was the best treatment for them^43^, ^44^, ^45^), and their reported adherence to SM/ERC may have been further influenced by the knowledge that they were taking part in a research study. Hence, future research could consider the perspectives and experiences of nontrial participants, and target individuals from minority ethnic groups.

This qualitative study was designed to support interpretation of the trial's primary outcome; hence, the questioning and analysis focussed on QoL experiences in relation to treatment allocation. However, it is possible that an individual's level of frailty (observed or self‐assessed) may also impact their experience of SM/ERC; this is another area where future (qualitative) research may be beneficial.

## 5 CONCLUSION

Our findings offer important insights that can be used to support informed decision‐making about SM/ERC management for dens fractures in older/frail patients where, increasingly, it is being recognised that optimising QoL should be the paramount consideration. Study findings also highlight the importance of clear information and comprehensive health and social care provisioning to optimise the gains and minimise adverse impacts of SM/ERC. Our study illustrates that the QoL benefits and drawbacks to SM/ERC can vary between individuals and may be influenced and informed by a variety of issues, including their personal circumstances and pre‐existing comorbidities and frailties. Hence, our findings underscore the importance of providing individualised patient‐centred care. To achieve this, it is vital that people are given comprehensive information about all these potential benefits and drawbacks to help them determine the treatment option best suited to them and their personal circumstances.

## AUTHOR CONTRIBUTIONS

**Mia Closs**: Methodology; investigation; writing—original draft; writing—review and editing; formal analysis; data curation. **Paul Brennan**: Conceptualisation; writing—review and editing; funding acquisition; investigation. **Angela Niven**: Writing—review and editing; project administration; investigation; data curation. **Susan Shenkin**: Conceptualisation; writing—review and editing; funding acquisition. **Helen Eborall**: Methodology; investigation; writing—review and editing; formal analysis; supervision. **Julia Lawton**: Conceptualisation; methodology; writing—review and editing; formal analysis; validation; supervision.

## CONFLICT OF INTEREST STATEMENT

The authors declare no conflicts of interest.

## ETHICS STATEMENT

Ethical review for this study was obtained as part of the trial application (REC ref: 21/SS/0036—Scotland; REC ref: 21/YH/0141—England). Full description included in Section 2.1 of paper. Informed consent provided by all interviewees.

## Supporting information

Supporting information.

Supporting information.

## Data Availability

Research data are not shared. Data will only be made available via direct request and only after completion of the associated clinical trial. ‘Permission to reproduce material from other sources’ is not applicable to this paper.
