# Supplementary material for: Understanding the quality‐of‐life experiences of older or frail adults following a new dens fracture: Nonsurgical management in a hard collar versus early removal of collar
Source: Health Expect. 2024 Mar 15;27(2):e14017. doi: 10.1111/hex.14017 (PMC10941537; doi:10.1111/hex.14017)
Supplement: Supplementary file 1 — Supporting information. [file HEX-27-e14017-s002.docx]

**Additional quotes/Illustrative data**

| **Domain** | **Theme** | **Quote/Illustrative data** |
| --- | --- | --- |
| Autonomy | Comparison with other health related restrictions/issues | [NAME]… has dementia. He has heart failure, he has osteoporosis, leukaemia, prostatitis, glaucoma, you name it… he’s got lots of health issues. So the neck has…not been an issue at all, it’s all his other problems. **D1_SM** |
|  | Needing additional help from others – dressing, shopping etc | Well, fortunately my wife helps me from time to time. I can do most of the basic things myself, but there’s one or two things where I can’t, for instance, I can’t always put my socks on, or stuff like that, you know. I can’t bend as I used to be able to bend. So sometimes she helps me put my socks on, you know. **D19_ERC** |
|  | Help with feeding | Obviously you can’t bend your neck forwards very much. And because she couldn’t see very well, she found eating difficult. So we used to go in and actually feed her. **D33_SM** |
|  | Hair/shaving | He looks like Father Christmas. Yeah, and his hair as well, because it comes up quite high at the back, em, we tried dry shampoo, but it doesn’t- it doesn’t do much really. But he can’t shave. And that- that aggravates him a bit. **D32_SM** |
|  | Problems with stairs | One thing that is- is difficult, with the collar on, that’s going up and down stairs - you can’t look down. … If you walk downstairs, you naturally look at them [yeah] you don’t keep your head up, do you, looking across the way. You know, you- you eh, and so going up and down stairs is a major event if you’ve got one of those collars on (laughs). **D11_ERC** (Talking about period of time wearing the collar) |
| Role and Activity | Not doing things normally enjoyed | *I've just not had the strength or the energy to be honest. And I didn't want to fall. Like my main thing, I mean I've not been down the back garden since [the fall in the garden] …. It's funny, it makes you lose your confidence… And I love going to feed the birds.* ***D16_ERC*** |
|  | Difficulties with craftwork etc | I’m an avid doku person, I’ve- I do a lot of patchwork and sewing and stuff like that. But there’s no way I could do that to bend over or sit long enough to do that. I’m not at the point yet where I’m ready to do that. I’m not- I know I wouldn’t be comfortable doing it. So there’s no point thinking about it. **D29_SM** |
|  | Hearing aids | You know, these things are one size fits all (laughs) and em, and the liner thing is- tended to cover the microphone up. … The trouble is when you rely on hearing aids, em, it’s difficult to go without- well I can go without them when I’m indoors, but you know, you get cut off. **D28_ERC** (Participant did not remove collar). |
|  | Feeling self-conscious | I felt a bit- well a bit embarrassed going anywhere, you know. Cause you think- you think I would care less, but you always had people staring at you, that sort of thing. … I felt I was a target, they all look at you as you walk away. **D9­_SM** |
| Relationships | Reversal of caring role/concern for dependants | *I mean me husband he’s really wobbly on his legs with this balance problem, so I have to be his carer. But he’s caring for me at the moment. So I don’t know what’ll happen, but we’re a’right.* ***D16_ERC*** |
|  | Family being protective | But my husband you know, he’s so protective, he’s like: right. That’s enough. I’ll do the rest. I’ll do the rest (laughing). But he kind of goes over the top a bit, you know. Why are you standing up? Sit down. I’m like: no! I’ve got to move around. **D21_ERC** |
|  | Experience of lack of help/support | *I mean, it was quite difficult. The Monday after he did it I went to our GPs and asked them…’cause he needed a frame ’cause the stick wasn’t strong enough…And I must admit, I broke down there because all they said to me was, go to social services. I ’phoned social services and they said to me, you’re on a list. And that was it.* ***D6_ERC*** |
|  | Care services not available | *We asked the local surgery to come and put a new liner in… but they didn’t come… absolutely nobody [came]. We’d gone to the surgery, we contacted all sorts of people, we even went through [charity working with older people]. Finally [charity] got her a nurse. … And the nurse walked in and said: where’s your wound. …She saw the collar and said: oh I’m not touching that, and left.* ***D10_SM*** |
|  | Experiencing good support and training | I mean the physio’s been great. And as I say, I’ve not been well the last week. So I only phoned her this morning, and she was gonna phone me back today, which she hasn’t. But that’s fine, I’ll phone back, and somebody ‘ll be in touch. So I’m not- I’m not concerned about that. **D29_SM** |
| Emotional Comfort | Heightened awareness of vulnerability | I seem to have developed a fear of balancing after that fall. I’m no dizzy, just a bit wary, you know. Apprehensive, very careful, careful. I just felt unsteady and eh, insecure. Not in control. That’s the best way of putting it. It’s [the fall] the first sort of major thing I’ve had in my life. **D9_SM** |
|  | Emotional impact of increasing disability | He’s really upset with himself, he keeps crying ’cause he’s really upset with himself for being like he is. And I think he’s just fed up with being in pain. **D6_ERC** |
|  | Concern about lack of information or guidance | I’m just worried if he hasn’t got this collar for protection, and when I was watering the garden, he was out there, and he wanted to water the garden, you know. And I- I felt if he swung about- I don’t know how- nobody’s told me how to- if- what he can do, and what he can’t do. **D14­_ERC**  (Spouse joining in conversation) |
|  | Positive/negative feelings about the collar | I was absolutely just appalled at how horrible the collar was. And it was almost a feeling of claustrophobia. A couple of nights and I was trying to go to sleep, and I was just feeling, I can’t stand this thing any more. You know, I hope I don’t get to the point where I just want to tear it off. **D2_ERC** |
|  | Worry if ERC is safe/effective | My main concern was, you know, this is research, I don’t want to damage my health any more than it’s already damaged. And envisaging myself with a broken bone in my neck without support was a little scary. **D2_ERC** |
| Attitude and Adaption | Practical home adaptions | But I’ve had a rail put in now. So that’s another thing, put a rail in the toilet. Or my builder did ’cause I didn’t want one of the cheap, plasticky things ’cause I’ve had a new bathroom so I wanted something a bit more decent. So I got stainless steel ones. **D6_ERC** |
|  | Benefit of adaptions | I- I’ve got two commodes. I’ve got a commode upstairs for the middle of the night. And I’ve got a commode downstairs so I don’t have to negotiate the steps every time I want to go for a wee. **D34_SM** |
|  | Different clothes/straws etc | (laughs) Em, drinking I’d put a napkin underneath that stops me making a mess. Em, and pick out the clothes that go over your head, or won’t go over your head, you button up down the front, so (laughing). **D28_ERC** (Participant did not remove collar) |
|  | Taking collar off | I hate it. Absolutely hate it (laughs) … But like now, I’m not wearing it, because I’m just sitting in the chair, just relaxing. But if I get up and do things, I- I put it on. … and I don’t sleep in it either eh, I usually get dressed first and then put the collar on. If I’m in the washing machine and, you know, cleaning and things like that, I tend to you know, to put it on. **D38_SM** |
|  | Not accessing collar again | No, never. I don't want to see the thing, it's out in the garage now. I thought my husband had binned it, I forgot to do it. I just think it's a shame that it can't be reused. It can't be sanitised and reused, that's my only desire for the collar. **D2_ERC** |
| Financial Security | Accessing support not otherwise available | We’ll see how that works, but em, if not I’m quite prepared to pay and get private physio. I’m done with all the nonsense now. **D29_SM** (Talking about waiting for an NHS physiotherapy referral) |
| Health Perception | Measuring/identifying improvement | It’s definitely on the mend. It’s not as sore as it was. … Before when it was- when I first did it [the fracture], it was really painful But it’s bearable now. **D21_ERC** |
|  | Pain relief from collar | he found great relief from it because of the pain in his neck, whenever I ask him about the pain in his neck, he says, oh he’s not feeling any pain in his neck. So, the collar, I think it’s doing its work in supporting his neck because it was hurting when he moved it before but he can’t move his head with the collar on. **D1_SM** |
|  | No pain relief from collar | I mean it did hurt with the collar - although it hurt without the collar – but it hurt more because it pressed. **D22_ERC** |
|  | No difference with/out collar | Well that’s something I was a bit disappointed that I still had quite a lot of pain when the collar came off, cause I thought it was going to be better. But that actually it wasn’t much better, em, with the collar off, than it was with the collar on. **D36_SM** (speaking about when the collar was removed at 6 weeks) |
|  | Rashes and sores from collar | I developed em, a very bad wound from the collar. You know, it rubbed my neck. and so the consultant at (names hospital) decided to take the collar off, and give the wound a chance to heal. So that’s what he did … That wound on my neck did heal. But it did take quite a long time. I’m still em, I’m still actually have got a dressing on it. **D36_SM** |
