# Supplementary material for: Understanding the quality‐of‐life experiences of older or frail adults following a new dens fracture: Nonsurgical management in a hard collar versus early removal of collar
Source: Health Expect. 2024 Mar 15;27(2):e14017. doi: 10.1111/hex.14017 (PMC10941537; doi:10.1111/hex.14017)
Supplement: Supplementary file 2 — Supporting information. [file HEX-27-e14017-s001.docx]

**Appendix 1 - Topic guide for patient/caregiver interviews**

*Baseline interview*

**Background information**.

- Can you tell me a bit about yourself [or your family member] e.g. what do you [or they] do and who you [or they] live with, what was your [or their] health like before the fracture?
- Any prior experiences of fractures and falls?
- Underlying health conditions, prior experiences of pain and pain management.
- What kinds of things were you [or they] able to do before the fracture? *Care packages?*
- How would you describe your [or their] quality of life at that time?

**If a caregiver** –

- Explore their caregiving roles and responsibilities. *Care packages?*
- Their own state of health (physical and mental health) – support for their health directly
- How caregiving responsibilities impact their own QoL.

**This fracture**

- Can you tell me a bit about what happened when you [or they] fractured your [or their] neck and how you [or they] ended up in hospital?
- How were you [or they] feeling at the time?
- What do you understand about the fracture – about fracturing your spine?
- What are your feelings about this fracture?
- How will it heal?
- What do you need to do to get better?
- How are you getting on at the moment?
- How would you describe your QoL just now?

**The recruitment approach**

- At what point did someone approach you about the trial and what are your recollections of what you were told?
- Probe to get people’s views about the timing of the approach; how information about the trial was delivered and what might be improved?

**Their understanding of the trial**

- What were your thoughts and impressions of the trial?
- What do you think the trial’s purpose is?
- Randomisation - Probe to explore understandings of randomization
- Do they feel they understood the trial etc.

**If carer –**

- - Do they think pt understood?
  - What do they base that on?

**Decision**

- Why did you agree to take part [or give agreement for family member to take part]?
- Did you need to take a bit of time to make your decision?
- Was anyone else involved in the decision, and why?
- Dynamics of decision making with them in past

**If carer –**

- - Do they think pt understood?
  - What do they base that on?
  - Decision making dynamics in past?

**Preference**

- Did you have any strong views about which treatment you [or they] would get.
- How did you feel when you found out you [or they] have been allocated to [control/intervention]?
- Reasons for any worries or concerns

**Our Learning**

- What can we learn from you experiences of trial recruitment which might help us to recruit other people?

**What happens next?**

- Explore participant’s understanding of what will happen next i.e. over the next 12 weeks.
- If randomized to the no collar arm – explore how they feel about this, any worries or concerns about not using a collar?
- If randomized to standard care – do they envisage any challenges or difficulties with keeping the collar on, and why. Any help or support they will need to keep the collar on?

Is there anything else you would like to add?

Clarify the timing and purpose of the follow-up interview; and arrange a provisional date or time.

………………………………………………………………………………….

*Follow-up interview*

**What’s been happening? (Take it chronologically)**

- Can you tell me a bit about what it’s been like for you since we last spoke? What happened after you [or they] left hospital? (Remind them where the last interview left off)
- What was it like when you first got home? How were you feeling at that point?
- Can you take me through a typical day?

**Info and advice from hospital?**

- What information and instruction were you given about how to manage the fracture?
- How easy has it been to follow this in practice?
- Reasons for following/not following instructions
- Use of pain medications etc.

*If in the intervention arm,*

- Any attempt made to access and use a collar, and why?
- Involvement of health professionals/others in that decision-making

*If in standard care arm*

- Reasons for the collar being removed,
- How often and what precipitated the decision to remove collar e.g., pain, discomfort, poor sleep.
- Was collar removal discussed with health professionals/others?
- Were attempts made to not disclose collar removal to others, and why?
- What might help you/other people to keep the collar on?

**Perceived impact of wearing/not wearing the collar**

- On comfort and pain.
  - What was the pain, neck mobility and discomfort like? (Describe).
  - Has this changed over time?
- On quality of life
- Ability to undertake everyday activities?
- *Care packages*

**Preparedness**

- On reflection, how prepared/aware do you think you were that tx and recovery might be like this?
- What would have helped you, if you were back at day one again?

**Contact and support**

- What has been received from health professionals? (Hospital/trial/community)
- Care packages?
- How this might be improved?

**Trial paperwork**

- Ease of understanding, remembering/prompts and completion
- Did they do it
- Are the questions capturing what is important to you?
- What are they missing?

**Retrospective preference**

- If you had your time again, would you have preferred to have been in the other arm of the trial?
- What can we learn from your experience that would help people in future?
